# The potential impact of artificial intelligence in radiology

**DOI:** 10.1590/0100-3984.2017.50.5e1

**Published:** 2017

**Authors:** Omir Antunes Paiva, Luciano M. Prevedello

**Affiliations:** 1 MD, Radiologist, Hospital Israelita Albert Einstein, São Paulo, SP, Brazil. E-mail: omir@outlook.com.; 2 MD, MPH, Neuroradiologist, Head of the Division of Medical Imaging Informatics, The Ohio State University Wexner Medical Center, Columbus, OH, USA. E-mail: luciano.prevedello@osumc.edu.

Artificial intelligence (AI) will bring changes to the professional life of radiologists, just as it has modified many other aspects of our lives. Since the invention of electricity, the internet, and most recently AI, general purpose technologies have made it possible for societies to progress and improve their quality of life.

Radiologists are not the only, or even the first, professionals to have their specialty modified by AI. Other areas of medicine have also been thus affected. Recently, one group of researchers developed software for cell phones and conducted a study in which the software was shown to distinguish malignant melanomas from benign lesions more accurately than did the dermatologists participating in the study^(1)^. Although the role of the dermatologist is much broader than the classification of lesions, that finding could be an initial indication that AI will also modify the labor market in that field.

Currently, the vast majority of laboratory tests are analyzed in automated systems, which does not seem to bother anyone. The need for trained personnel to quantify red blood cells has long since ceased to exist. Likewise, advances in the field of computer vision have begun to produce improved sensitivity in the detection of tumor in histopathological specimens when compared to human analysis^(2)^.

The main factors that allowed such advances in artificial intelligence were the abundance of data, the development of artificial neural networks, and the increased affordability of the hardware:

- Currently, data are collected by various instruments. If AI is the new electricity, data is the new coal.
- The development of artificial neural networks allowed some of the problems with other machine learning techniques to be solved. In traditional methods, which are the basis of some auxiliary diagnostic software—computer-aided detection—increasing the amount of data improves accuracy to a certain extent. In the deep learning technique, which uses hidden layers of artificial neural networks, the accuracy tends to continue to increase as new data are added. For example, in 2015, in an annual competition of object recognition in everyday images, the performance of a machine surpassed that of a human for the first time^(3)^. Deep learning is a branch of machine learning, which is in turn one of the branches of AI.
- The increased affordability of hardware, notably processing and storage devices, also fostered advancement in the area. Currently, one can purchase a laptop with the same processing power as the most powerful supercomputer manufactured in the year 2000, the difference being that the laptop will cost approximately 40,000 times less than the 110 million dollars spent on the supercomputer and will weigh much less than its 106 tons^(4)^.

Advances in the field of computer vision have aroused the interest of technology giants and various government sectors in highly lucrative areas of great global interest. Autonomous cars and drones with automatic target recognition require instantaneous processing of images, do not tolerate errors, and are not supposed to rely on a human who is readily able to modify their operation. Although this dispersion could apparently cause a delay in the influence of AI in the radiology market, advances in the automotive and space sectors are commonly catalysts for advances in other sectors, potentially accelerating the pace of new discoveries in the field of imaging.

The implementation of AI techniques in medical imaging has particular challenges. Diagnoses are not always confirmed; classifications and concepts are not always unanimous, nor are they eternal^(5)^. The structures of the human body present great variation in terms of their normal dimensions and textures, such variation potentially masking pathological conditions.

Knowledge regarding the automated analysis of medical images has spread rapidly. One example is provided by the most recent Data Science Bowl^(6)^, which offered a total of a million dollars in prizes for teams that developed and made available the best automated nodule classification algorithms for chest computed tomography (CT). Several participating teams had never worked in the field of medical imaging, and many of them likely chose to work in the field only because of the competition.

The impact of AI on the routine of the radiologist will probably occur gradually. Software will provide data that we cannot extract from the images, prioritize examinations according to disease severity^(7)^, and, among other resources, will gradually become part of the routine. In certain areas of radiology, AI has already proven capable of generating parts of the radiological report, including a preliminary description of the imaging findings and the measurement of some lesions. However, the main contribution of the radiologist is not simply to provide this information but to integrate it with the clinical data, contributing in a more holistic way to the diagnosis and individualized treatment of the patient. This type of information integration will take more time to be replicated by machines. Nevertheless, radiologists who know how to use the technology in their favor will have a clear advantage over those who resist it. Reducing the time required for reading images can provide more time for direct patient care, thus allowing the radiologist to play a more effective diagnostic role by analyzing data from a variety of sources, rather than image-based data alone^(8)^. In addition, the general training of a radiologist goes beyond image interpretation, including the performance of minimally invasive procedures, quality control, and management, as well as engaging in multidisciplinary discussions and acting as a consultant (for the selection of the most appropriate imaging method to address a given clinical issue).

The market for radiology services has already undergone extreme changes due to technological advances. Although jobs have not ceased to exist, roles have been redefined. If we told radiologists 30 years ago that barium-based examinations would gradually fall into disuse, they would probably have been pessimistic about the job market in their field. However, the imaging methods that subsequently emerged made it possible to expand the market for radiology services. Radiologists who have not adapted to the changes and have not learned the new techniques have more difficulty in the current job market. Our specialty must adapt to the innumerable modifications that AI will make possible, among which will undoubtedly be advances that will improve the diagnosis and care of our patients.

## References

[1] Esteva A, Kuprel B, Novoa RA (2017). Dermatologist-level classification of skin cancer with deep
neural networks. Nature.

[2] Liu Y, Gadepalli K, Norouzi M Detecting cancer metastases on gigapixel pathology images.

[3] He K, Zhang X, Ren S Delving deep into rectifiers: surpassing human-level performance
on imageNet classification. https://arxiv.org/pdf/1502.01852.pdf.

[4] Oram A (2001). Peer-to-peer. Harnessing the power of disruptive technologies.

[5] Kohli M, Prevedello LM, Filice RW (2017). Implementing machine learning in radiology practice and
research. AJR Am J Roentgenol.

[6] Kaggle Data Science Bowl 2017.

[7] Prevedello LM, Erdal BS, Ryu JL (2017). Automated critical test findings identification and online
notification system using artificial intelligence in imaging. Radiology.

[8] Jha S, Topol EJ (2016). Adapting to artificial intelligence: radiologists and
pathologists as information specialists. JAMA.

